# White Light Enhances Adhesive Strength Between Epidermal and Inner Tissues of Pea Epicotyls via Accumulation of Cell Wall‐Bound *p*‐Coumaric Acid

**DOI:** 10.1111/ppl.70755

**Published:** 2026-01-25

**Authors:** Yuma Shimizu, Kazuyuki Wakabayashi, Kensuke Miyamoto, Kouichi Soga

**Affiliations:** ^1^ Department of Biology, Graduate School of Science Osaka Metropolitan University Osaka Japan; ^2^ Faculty of Liberal Arts, Sciences and Global Education Osaka Metropolitan University Osaka Japan

**Keywords:** adhesive strength, cell wall, epidermal tissue, inner tissue, *p*‐coumaric acid

## Abstract

The epidermal and inner tissues of stem organs adhere via the cell wall, and the adhesion of both tissues is involved in the structural integrity of the stem. We have developed a method to quantitatively measure adhesive strength between these tissues. In this study we examined the effects of white light on adhesive strength between epidermal and inner tissues of pea epicotyls, as well as the chemical properties of cell walls in both tissues. The irradiation of white light to etiolated seedlings resulted in significantly higher adhesive strength between epidermal and inner tissues, as well as inhibition of elongation growth of the epicotyls. Further observation of epicotyl cross‐sections revealed that white light substantially increased the intensity of autofluorescence emitted from the cell wall in epidermal tissue and the outermost layer of inner tissue, accompanied by color alteration. Overall, the spectrum of the autofluorescence emitted from light‐irradiated epicotyl sections matched that of reference standard *p*‐coumaric acid, a phenolic acid. Further chemical analysis of cell wall constituents revealed that cell wall‐bound *p*‐coumaric acid was predominantly accumulated in epidermal tissue in response to light irradiation. Taken together, these results suggest that light‐induced accumulation of cell wall‐bound *p*‐coumaric acid in epidermal tissue may enhance the adhesive strength between epidermal and inner tissues. Moreover, increased adhesive strength between these tissues may contribute to light‐induced inhibition of pea epicotyl elongation.

## 1 Introduction

Mechanical interactions between plant tissues can play a pivotal role in maintaining body shape and regulating plant growth (Kelly‐Bellow et al. 2023). Moreover, the role of epidermal and inner tissues in the regulation of elongation growth of stem organs has been intensively studied in dicotyledonous and gramineous seedlings (Kutschera and Niklas 2007). These studies have demonstrated that when segments excised from stem organs were separated into epidermal and inner tissues and incubated separately, the isolated epidermal tissue underwent contractions immediately after peeling. In contrast, the isolated inner tissues (i.e., the peeled segments) exhibited greater elongation than unpeeled (intact) segments (Masuda and Yamamoto 1972; Kutschera et al. 1987; Wakabayashi et al. 1991, 1999). Taken together, these findings indicate that epidermal tissue from stem organs is subjected to strong tissue tensions produced by expanding forces from inner tissues. As a consequence, elongation of stem organs is regulated by the degree of extension of epidermal tissue. In addition to the role of epidermal and inner tissues in regulating stem growth, it has been hypothesized that adhesion of epidermal and inner tissues may influence stem growth since both tissues adhere firmly via the cell wall. We have recently developed a method to quantitatively measure the adhesive strength between the epidermal and inner tissues of pea epicotyls (Shimizu et al. 2025). In that study, we demonstrated that the adhesive strength between both tissues in the elongating region of the epicotyl was significantly lower than in non‐elongating regions.

Since plants are subjected to a variety of environmental stimuli, including light, temperature, and water, they modify growth and development patterns to facilitate environmental adaptation (Bar and Ori 2014; Waadt et al. 2022). Light, in particular, is a pivotal stimulus that influences the growth and morphogenesis of plant organs. For example, excessive stem elongation of etiolated (dark‐grown) seedlings is known to be rapidly suppressed upon exposure to light irradiation (Miyamoto et al. 1992; Symons and Reid 2003). Light has been found to reduce the mechanical extensibility of the cell wall in the stems of various plants, including cucumber and sunflower hypocotyls, pea epicotyls, and maize and rice coleoptiles (Cosgrove 1988; Kutschera 1990; Kigel and Cosgrove 1991; Miyamoto et al. 1992; Tan et al. 1992a; Parvez et al. 1996). Moreover, this effect has been found to be concomitant with inhibition of stem growth.

In addition to mechanical properties, light irradiation has been found to modify the chemical properties of cell walls in stem organs. For example, sunflower hypocotyls have been shown to increase the thickness of the outer epidermal cell wall in response to exposure to white light (Kutschera 1990). In another study, Miyamoto et al. (1997) showed that white light irradiation increased the molecular mass of xyloglucans in the cell walls of pea epicotyls. This increase was associated with a reduction in the mechanical extensibility of the epicotyl cell wall. Moreover, exposure to white light has been found to suppress the turnover of (1,3),(1,4)‐β‐glucan in the cell walls of rice coleoptiles by reducing (1,3),(1,4)‐β‐glucan hydrolase activity (Chen et al. 1999). Beyond polysaccharide components, light irradiation has been shown to increase the content of lignin, a high‐molecular‐mass phenolic compound in the cell walls of various plants (Moura et al. 2010; Gall et al. 2015). Furthermore, cell wall‐bound phenolic acids such as ferulic acid, *p*‐coumaric acid, and diferulic acid in the cell walls of rice and maize coleoptiles are known to increase significantly in response to light exposure (Tan et al. 1992a; Parvez et al. 1997). Moreover, this effect is accompanied by a decrease in the mechanical extensibility of the cell wall. Overall, these findings suggest that light irradiation may affect the adhesive strength between the epidermal and inner tissues by modifying the chemical properties of the cell wall. This modification may, in turn, inhibit stem organ elongation.

In this study, we aimed to elucidate the effects of white light irradiation on the adhesive strength between the epidermal and inner tissues of the second internode region of pea epicotyls. Furthermore, we analyzed differences in the chemical properties of the cell walls of both tissues that were induced in response to light irradiation by optical observation using a microscope and chemical analysis of cell wall constituents. As a result, we found that white light irradiation induced a substantial accumulation of cell wall‐bound *p*‐coumaric acid in epidermal and adjacent inner tissues of epicotyls. This accumulation may enhance tissue adhesion and restrict elongation by limiting inner tissue expansion.

## 2 Materials and Methods

### 2.1 Plant Materials and Growth Conditions

Pea seeds ( *Pisum sativum* L. cv. Alaska) were purchased from the Watanabe Seed Corporation. For germination, the seeds were first soaked in running tap water for 1 day at 20°C before being germinated on a plastic dish filled with water kept at 25°C in the dark. After 5 days, we selected seedlings with a 30–35‐mm‐long second epicotyl internode and marked the top 5 mm of the upper region (i.e., 5–10 mm below the hook tip) using India ink. Marked seedlings were randomly selected and cultivated either in darkness or under white fluorescent light (FLR40S·W/M; Panasonic) at an intensity of 40 μmol m^−2^ s^−1^ (measured at the plant level) for 24 h at 25°C. After incubation, the length of the marked region was measured using a ruler. All manipulations were performed under dim green light (i.e., 0.09 μmol m^−2^ s^−1^ at the handling level).

### 2.2 Strength of Adhesion Between the Epidermal and Inner Tissues

After incubation of the seedlings in darkness or light for 24 h, we excised a 20‐mm‐long segment (i.e., 5–25 mm below the hook tip) from the second internode. Next, we measured the adhesive strength between the epidermal and inner tissues using a method described by Shimizu et al. (2025). Briefly, the edge of partially peeled epidermal tissue (the “peeling arm”) and the remaining segment region were fixed to the upper and lower clamps (Fiber filament tensile grips 2711–006; Instron) of a tensile tester (Tensilon STB‐1225S; A&D). Epidermal tissue (~10 mm long) was then peeled from the epicotyl segment by raising the upper clamp at a speed of 100 mm min^−1^. We recorded the force required to peel the last 5 mm of the ~10 mm length; these values were averaged and defined as the peeling force. After these measurements, we measured the width of the peeled epidermal tissue segment using a microscope. Finally, we defined the peeling force normalized by the width of the peeled epidermal tissue as the adhesive strength.

### 2.3 Cell Wall Autofluorescence

After incubating seedlings under darkness or light for 24 h, 5 mm‐long segments (sampled 5–10 mm below the hook tip) were excised from the second internode. Epicotyl segments were then fixed in 4% (w/v) paraformaldehyde in 0.1 M phosphate buffer (pH 7.2) (Fujifilm Wako Pure Chemical Corporation). Next, fixed segments were embedded in 5% (w/v) agar, after which 150 μm cross‐sections of the middle part of the fixed segments were cut using a vibratome (VT1000S; Leica Biosystems). Cross‐sections were observed under a fluorescence microscope (Axio Imager. A1; Carl Zeiss) under UV excitation at a wavelength of 365 nm. Autofluorescence was detected using a 420 nm long‐pass filter, and images were captured using a digital camera (DP74; Olympus).

### 2.4 Lignin Staining

Lignin staining was conducted according to the method described by Wakabayashi et al. (2009). Briefly, phloroglucinol reagent was prepared by mixing two volumes of 2% (w/v) phloroglucinol (Fujifilm Wako Pure Chemical Corporation) in 95% (v/v) ethanol with one volume of 30% (v/v) HCl. Cross‐sections were then prepared from the second internode, as described above, before being stained with phloroglucinol reagent at room temperature and observed under a light microscope (Axio Imager. A1; Carl Zeiss). Bright‐field images were then captured using a digital camera (DP74; Olympus).

### 2.5 Autofluorescence Spectra

Autofluorescence spectra of cross‐sections prepared from the second internodes were analyzed according to the method described by Zhou et al. (2023). Briefly, the autofluorescence spectrum emitted from the portion of the cell wall between the epidermal tissue and the outermost layer cells of inner tissue was imaged using a confocal laser scanning microscope (TCS SP8; Leica Microsystems) and the data were analyzed using the lambda scan software (Leica Microsystems). Cross‐sections were observed under UV excitation at a wavelength: 405 nm. Autofluorescence emissions were detected from 450 to 540 nm using a detection step size of 3 nm, and spectra emitted from 10 cross‐sections obtained from 10 independent second internodes were subjected to fluorescence intensity normalization, after which the spectra were merged. To obtain autofluorescence spectra of reference standard phenolics, including *p*‐coumaric acid, ferulic acid, and coniferyl alcohol (Fujifilm Wako Pure Chemical Corporation), the phenolics were dissolved in 50% (v/v) methanol to reach a concentration of 50 mg mL^−1^. All phenolics were then recrystallized on glass slides, and the autofluorescence spectra of five independently prepared samples were analyzed using the method described above. Principal component analysis (PCA) of autofluorescence spectra was performed in the R software, with each normalized spectrum treated as an observation and each wavelength as a variable, following the method described by Karimali et al. (2020).

### 2.6 Fractionation of Cell Wall Components

After the incubation of seedlings in darkness or light for 24 h, 5 mm‐long segments (sampled 5–10 mm below the hook tip) were excised from the second internode. The quantification of the cell wall components was conducted using twenty segments for each sample. Epidermal tissue was then carefully peeled from the segment using fine forceps. Peeled epidermal tissue and remaining inner tissue were separately fixed in 80% (v/v) ethanol then stored at 4°C. Cell wall components of epidermal and inner tissues were fractionated according to Wakabayashi et al. (2015), as illustrated in Figure S1. After rehydration, epidermal and inner tissues were first homogenized in water, washed successively in water, acetone, and a 1:1 methanol:chloroform mixture (v/v). Samples were then treated with porcine pancreatic α‐amylase (2 units mL^−1^, type I‐A, Sigma) in 50 mM sodium acetate buffer (adjusted to pH 6.5). Ester‐linked phenolic acids were extracted from cell wall preparation using NaOH (4% w/v, 1 M) for 24 h at room temperature in the dark. The residual cell wall material was then extracted three times (8 h each) with 17.5% (w/v) NaOH containing 0.02% (w/v) NaBH₄. After extracting cell wall‐bound phenolics in 4% NaOH (see below), the remaining solution was combined with the 17.5% NaOH extracts, which had been neutralized with acetic acid, to obtain the matrix polysaccharide fraction. The alkali‐insoluble residue, representing the cellulose fraction, was washed with 0.03 M acetic acid and ethanol, dried at 40°C, dissolved in 72% (v/v) sulfuric acid, and diluted 29‐fold with water. The sugar content of both fractions was determined using the phenol–sulfuric acid method (DuBois et al. 1956) with glucose as the standard.

### 2.7 Cell Wall‐Bound Phenolic Acids

Analysis of cell wall‐bound phenolic acids was carried out according to the method described by Wakabayashi et al. (2015). First, ester‐linked phenolic acids were released from the cell wall with 4% NaOH as described above. The NaOH extracts were acidified to pH 3 using HCl and then extracted three times using ethyl acetate. The ethyl acetate extracts were combined then dried. The liberated phenolic acids were analyzed using an HPLC (LC‐6A; Shimadzu) equipped with an Inertsil ODS‐3 column (4.6 × 250 mm, GL Science Inc.) and a photodiode array detector (SPD‐M20A; Shimadzu). Samples were eluted using a linearly increasing concentration of methanol (10%–73%) in 5 mM trifluoroacetic acid (approximately pH 4). Phenolic acid monomers were then identified and quantified using *trans*‐ferulic acid and *trans*‐*p*‐coumaric acid as standards (Fujifilm Wako Pure Chemical Industries). The observed peak of diferulic acid was identified and quantified using 5–5′–diferulic acid that was synthesized using the method described by Richtzenhain (1949) and compared to published spectra and response factors reported by Waldron et al. (1996). During HPLC analysis, the quantification of phenolic compounds was feasible when the applied sample solution contained a minimum of 0.2 ng of each phenolic acid.

### 2.8 Statistical Analyses

The means and standard errors (SEs) were calculated for elongation, adhesive strength, and the amounts of cell wall polysaccharides and phenolics. Statistical significance was assessed using Welch's *t*‐tests implemented in the Microsoft Excel software. For PCA, the scores of the first two principal components (PC1 and PC2) were subjected to multivariate analysis of variance (MANOVA) in the R software, using Pillai's trace as the test statistic (Karimali et al. 2020). Subsequently, one‐way analysis of variance (ANOVA) was applied to the PC1 scores to assess group differences, followed by Tukey–Kramer multiple comparison tests for pairwise comparisons. *p* < 0.05 were considered statistically significant.

## 3 Results

### 3.1 Epicotyl Growth and Adhesive Strength Between Epidermal and Inner Tissues

**FIGURE 1:**
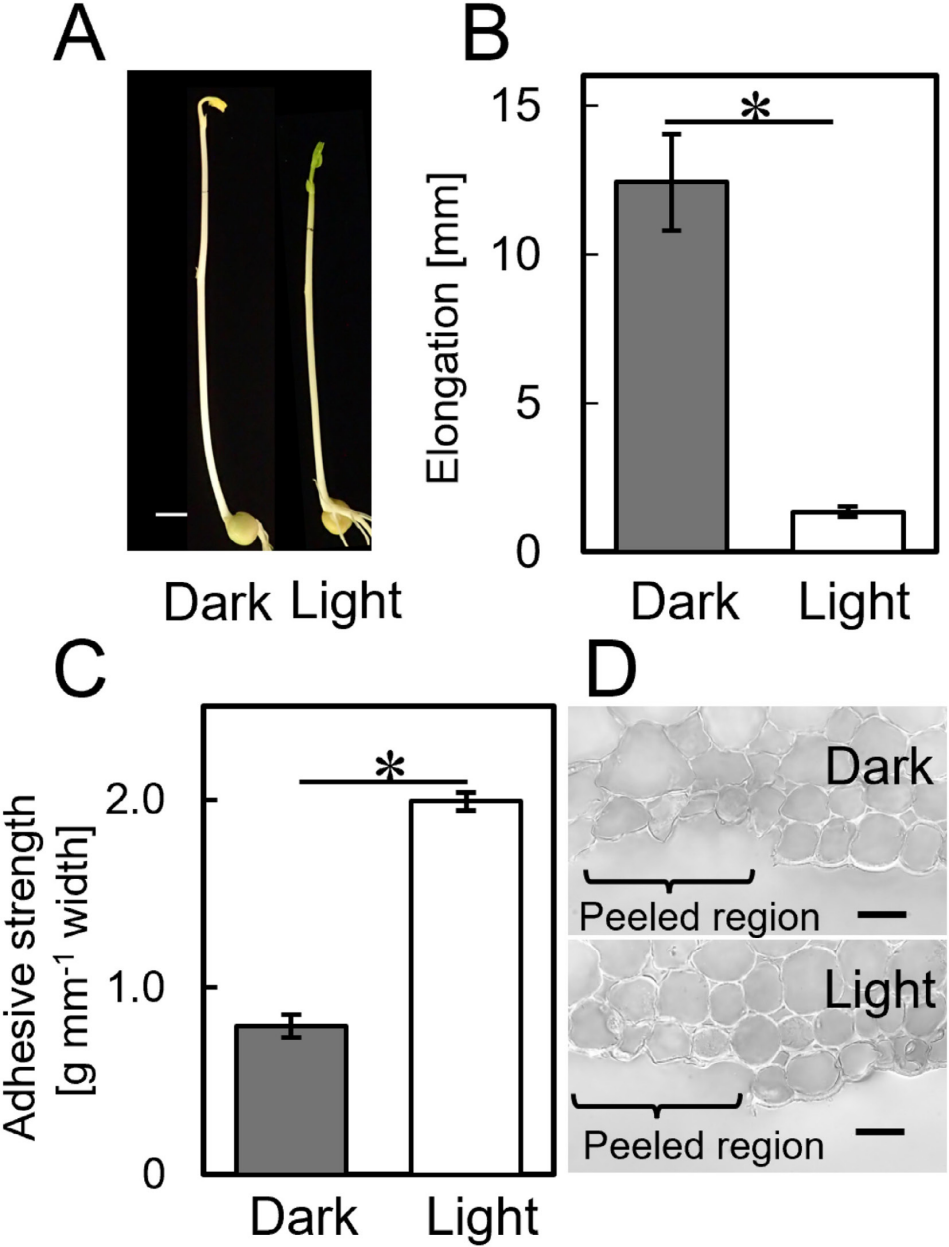
Effects of light irradiation on elongation and the adhesive strength between epidermal and inner tissues in pea epicotyls. Five‐day‐old etiolated pea epicotyls were grown in the dark or light for 24 h. (A) Representative photograph of dark‐grown and light‐irradiated pea seedlings. (B) Elongation growth of the marked region of the second internode during 24 h incubation. (C) Adhesive strength between epidermal and inner tissues. (D) Magnified view of the region where the epidermal tissue peeled off. Scale bars indicate 10 mm in (A) and 30 μm in (D). Values in (B, C) indicate mean ± SE (*n* = 10). Asterisk indicates significant differences in mean values (Welch's *t*‐test, *p* < 0.05).

Following a 24 h exposure to white light, we observed remarkable changes in pea seedlings, including hook opening and the onset of greening in the first leaves (Figure 1A). The marked region of the second internode of the epicotyl, which was initially 5 mm in length, elongated by approximately 12 mm during a 24 h incubation in the dark (Figure 1B). However, elongation of the marked region was suppressed by 90% under white light irradiation. The adhesive strength between the epidermal and inner tissues of the second internode is shown in Figure 1C. We found that the irradiation of white light for 24 h resulted in a substantial increase in adhesive strength. Moreover, images of epicotyl cross‐sections revealed that the epidermal tissue was detached as a single layer from the underlying inner tissue under both cultivation conditions (Figure 1D).

### 3.2 Autofluorescence Imaging and Lignin Staining

**FIGURE 2:**
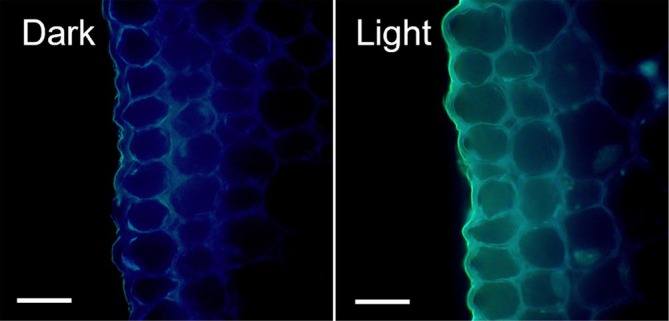
Effects of light irradiation on cell wall autofluorescence in pea epicotyls. Epicotyls were grown as described in Figure 1. Autofluorescence images of cell walls from cross‐sections of the second internode were observed under a fluorescence microscope under UV excitation (wavelength: 365 nm). Scale bar in the photograph indicates 30 μm.

**FIGURE 3:**
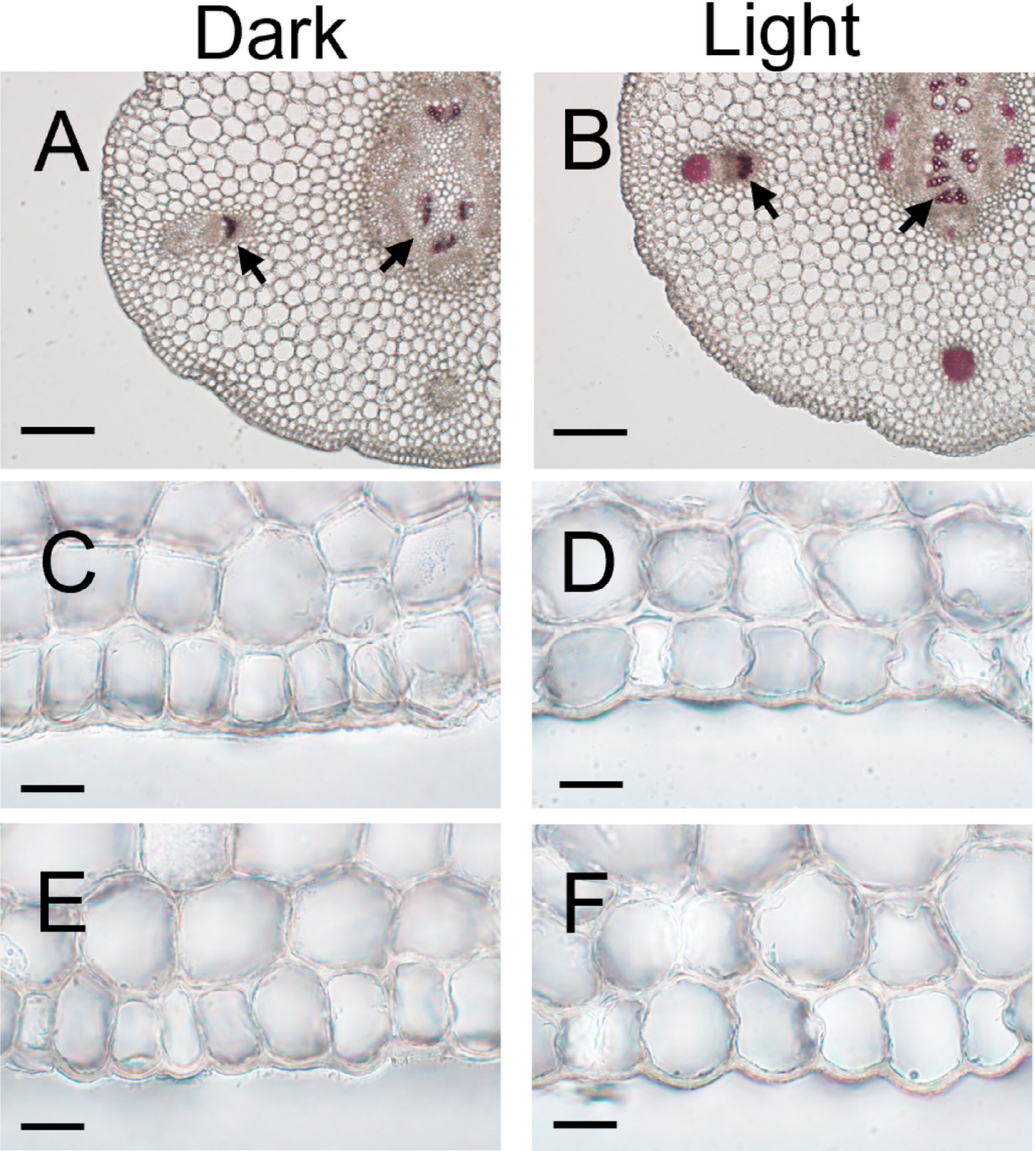
Effects of light irradiation on lignin deposition in pea epicotyls. Epicotyls were grown as described in Figure 1. Cross‐sections stained with (A–D) or without (E, F) phloroglucinol reagent. Scale bars indicate 200 μm (A, B) and 20 μm (C–F). Arrows in (A and B) indicate stained vascular bundles and xylem vessels.

Overall, the autofluorescence intensity emitted from the epidermal tissue and the outermost layer of the inner tissue at the second internode in dark‐grown epicotyls was weak, whereas the intensity in light‐irradiated epicotyls was significantly stronger and showed a marked alteration in color tone (Figure 2). These results suggest that light exposure induced the accumulation of cell wall components exhibiting autofluorescence in both tissues. Moreover, since the autofluorescence of plant cell walls is largely attributed to lignin, we examined the distribution of lignin using phloroglucinol reagent. As shown in Figure 3A,B, we detected pronounced lignin staining in vascular bundles and xylem vessels located within the inner tissues of the second internode in both dark‐grown and light‐irradiated epicotyls. However, lignin staining was not discernible in the cell walls of epidermal tissue and in the outermost layer of the inner tissue, even in light‐irradiated epicotyls (Figure 3C,D), as in unstained ones (Figure 3E,F).

**FIGURE 4:**
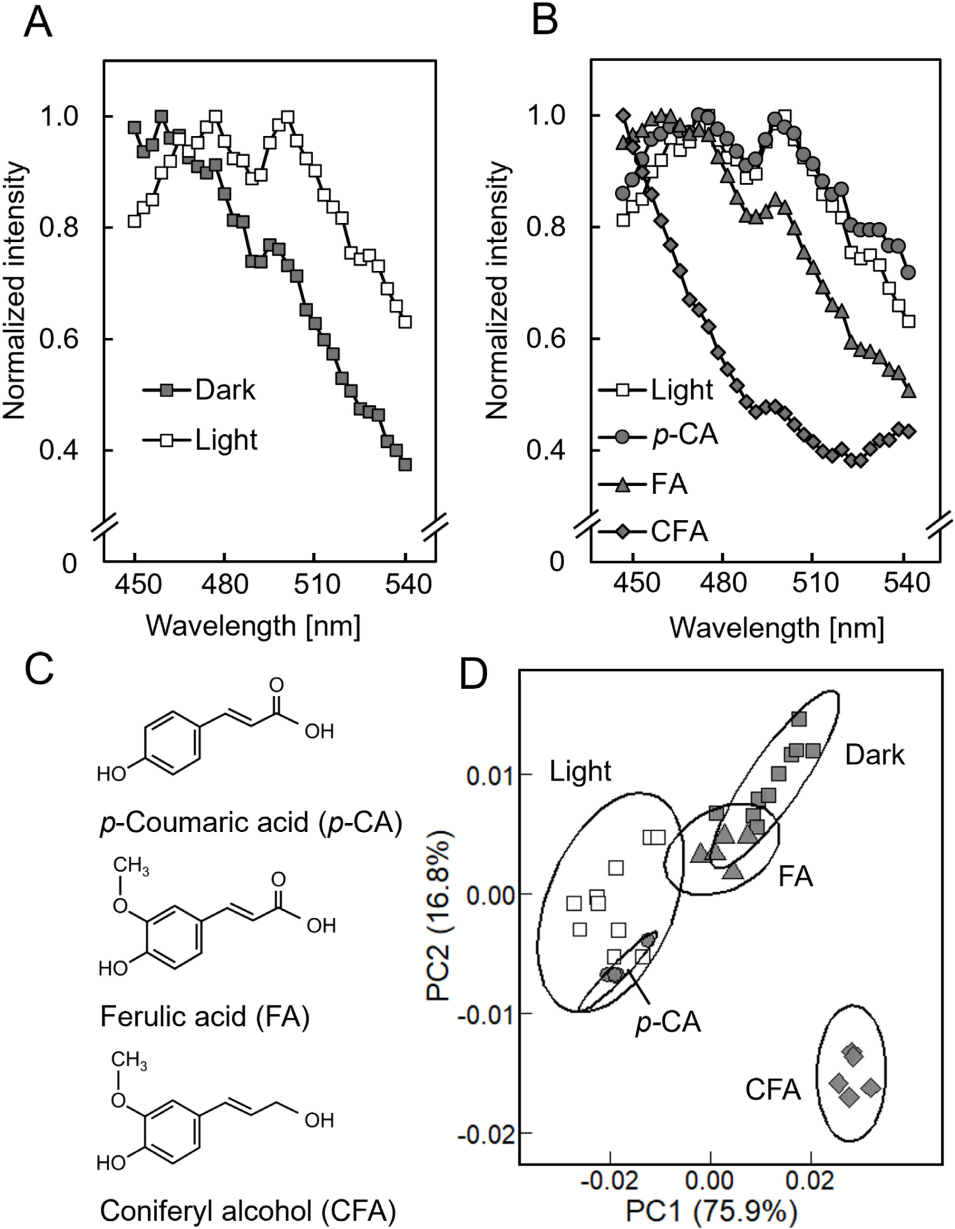
Autofluorescence spectra of cell walls in cross‐sections of pea epicotyls and authentic phenolics. Epicotyls were grown as described in Figure 1. (A) Autofluorescence spectra obtained from the portion of the cell wall between epidermal tissue and the outermost layer cells of inner tissue of the second internode of pea epicotyls. Spectra emitted from 10 cross‐sections obtained from 10 independent second internodes were merged. (B) Autofluorescence spectra obtained from light‐irradiated cell walls and reference standard *p‐*coumaric acid (*p‐*CA), ferulic acid (FA), and coniferyl alcohol (CFA). Phenolics were recrystallized on glass slides, and spectra from five independently prepared samples were merged. (C) Chemical structure diagrams of *p‐*CA, FA, and CFA. (D) Principal component analysis (PCA) score plot of autofluorescence spectra. The maximum value of the autofluorescence intensity in (A, B) was adjusted to 1.0.

Next, we examined autofluorescence spectra emitted from the portion of the cell wall between the epidermal tissue and the outermost layer cells of inner tissue (Figure 4). We found that the autofluorescence spectra obtained from epicotyls grown in the dark exhibited a gradual decrease from wavelengths of 450 nm to 540 nm (Figure 4A). In contrast, spectra obtained from light‐irradiated epicotyls differed from those of dark‐grown epicotyls. These spectra displayed two peaks at 474–480 and 498–504 nm. To elucidate the chemical nature of these signals, we examined the autofluorescence spectra of reference standard *p*‐coumaric acid, ferulic acid, and coniferyl alcohol. This revealed that the spectra obtained from light‐irradiated epicotyl samples were almost identical to the spectrum observed for *p*‐coumaric acid (Figure 4B,C).

To quantitatively evaluate differences in autofluorescence spectra, we performed a PCA. PC1 accounted for 75.9% of the total variance and PC2 for 16.8%, together explaining 92.7% of the variance (Figure 4D). A MANOVA based on PC1 and PC2 scores revealed a highly significant overall effect, indicating that spectral profiles differed substantially among dark‐grown samples, light‐grown samples, and phenolic standards. Because PC1 captured the majority of variance, we conducted a one‐way ANOVA on PC1 scores, which confirmed significant differences among groups. Tukey's test for PC1 showed no significant difference between light‐grown samples and *p*‐coumaric acid (*p* = 0.99), whereas all other pairwise comparisons were significant. These results indicate that the autofluorescence spectra of light‐grown epicotyl samples are highly similar to those of *p*‐coumaric acid.

### 3.3 Cell Wall Polysaccharide and Cell Wall‐Bound Phenolic Acid Content

**FIGURE 5:**
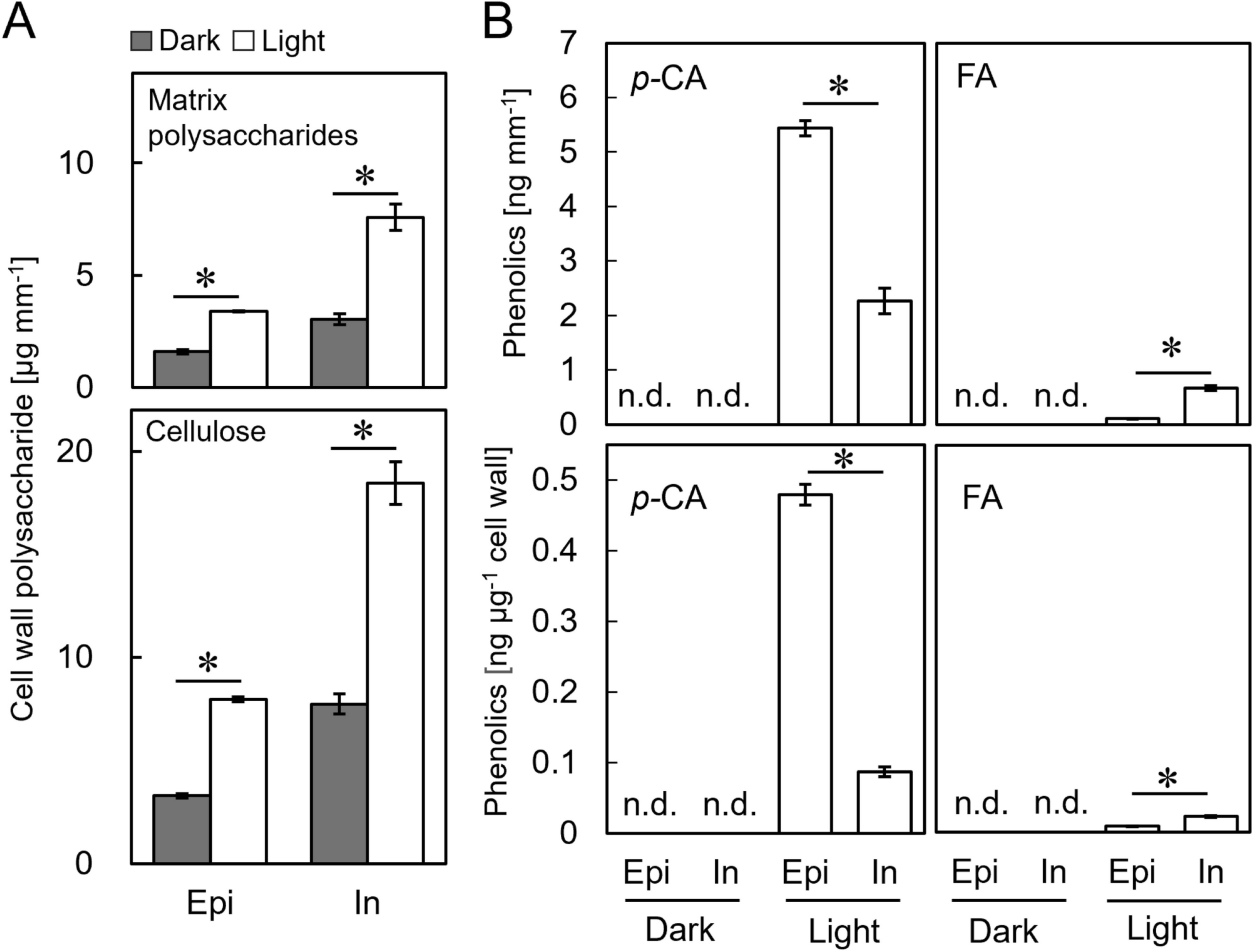
Effects of light irradiation on the contents of cell wall polysaccharide and phenolic acids in pea epicotyls. Epicotyls were grown as described in Figure 1. The quantification of the cell wall polysaccharide and phenolic acids was conducted using twenty segments for each sample. (A) Cell wall polysaccharide content. Epi, epidermal tissue; In, inner tissue. Data are shown as mean ± SE (*n* = 3). Asterisks indicate significant differences between mean values of the dark and light conditions (Welch's *t*‐test, *p* < 0.05). (B) Cell wall‐bound *p‐*coumaric acid (*p‐*CA) and ferulic acid (FA) content. Data are shown as mean ± SE (*n* = 3). Asterisks indicate significant differences between mean Epi and In values (Welch's *t*‐test, *p* < 0.05).

Next, we conducted a chemical analysis of cell wall constituents using isolated epidermal and inner tissue samples obtained from second internode segments. First, cell wall polysaccharides were fractionated into two fractions, that is, matrix polysaccharides and cellulose. The matrix polysaccharide and cellulose content of the epidermal and inner tissue samples, normalized by unit epicotyl length, is shown in Figure 5A. We also observed that all tissues sampled from dark‐grown epicotyls showed that cellulose content was almost twice that of matrix polysaccharides. Moreover, the irradiation of white light for 24 h significantly increased cellulose and matrix polysaccharide content in both tissues (Figure 5A). This result suggests that light exposure leads to cell wall thickening.

The cell wall‐bound *p‐*coumaric acid and ferulic acid content in both epidermal and inner tissue samples from second internode segments is shown in Figure 5B. The phenolic acid content was below the detection limit (i.e., under 4 pg μg^−1^ cell wall for both phenolic acids) in all tissue samples sourced from dark‐grown epicotyls. Conversely, the content of both phenolic acids significantly increased in response to light irradiation. Moreover, we observed significant increases in the content of *p‐*coumaric acid relative to ferulic acid in both tissue samples. The distribution of phenolic acids in epidermal and inner tissues revealed that *p*‐coumaric acid content was considerably higher in the epidermal tissue than in the inner tissue, while ferulic acid exhibited the opposite tendency (Figure 5B). Finally, the cell walls of samples obtained from the second internodes of both dark‐ and light‐grown epicotyls did not contain detectable amounts of diferulic acid.

## 4 Discussion

The present results demonstrated that white light irradiation for 24 h significantly increased the adhesive strength between the epidermal and inner tissues of the second internode of pea epicotyls when it inhibited the elongation growth of the epicotyls (Figure 1). Our previous results showed that in etiolated pea epicotyls, the adhesive strength between epidermal and inner tissues in non‐elongating regions was significantly greater than in elongating regions (Shimizu et al. 2025). Thus, increased adhesive strength between epidermal and inner tissues may limit expansive deformation of the inner tissue. Moreover, it has also been shown that white light irradiation decreased the mechanical extensibility of the cell wall in epidermal and inner tissues of the elongating region of pea epicotyls during inhibition of epicotyl growth (Miyamoto et al. 1992). Kelly‐Bellow et al. (2023) found that mutant plants with a brassinosteroid deficiency exhibited a dwarfed phenotype, which the authors attributed to slow growth of epidermal tissue. Taken together, our findings suggest that increased adhesive strength between epidermal and inner tissues as well as decreased cell wall extensibility of both tissues may play a pivotal role in light‐induced inhibition of pea epicotyl elongation.

Plant cell walls contain a variety of phenolic compounds, including lignin and various phenolic acids. Moreover, autofluorescence emissions of these compounds have been observed under UV irradiation, with lignin exhibiting especially strong autofluorescence (Harris and Hartley 1976). Here we found that the intensity of autofluorescence emitted from the cell wall between the epidermal tissue and the outermost cell layer of the inner tissue increased dramatically in white light‐irradiated epicotyls (Figure 2). These findings indicate that light exposure induced the accumulation of cell wall components exhibiting autofluorescence in both tissues. Taken together, these results suggest that autofluorescent substances within the cell wall at the interface between the epidermal and inner tissues may play a role in modulating their adhesive strength. In contrast to our expectation, increased cell wall autofluorescence intensity was not attributable to the lignin content (Figure 3). Instead, it resulted from an increase in monomeric phenolic acid content, particularly *p*‐coumaric acid (Figures 4 and 5B). In general, the cell walls of gramineous plants contain only a trace amount of ester‐linked monomeric phenolic acids, such as *p‐*coumaric acid and ferulic acid (Harris and Hartley 1980; Hartley and Harris 1981). Indeed, the contents of cell wall‐bound *p‐*coumaric acid and ferulic acid in etiolated pea epicotyls were below the detection limit (Figure 5B). However, upon light irradiation, there was a substantial increase in *p*‐coumaric acid, rather than ferulic acid, which occurred predominantly in the epidermal cell wall (Figure 5B). A previous study revealed that the exogenous application of *p*‐coumaric acid to etiolated rice seedlings suppressed coleoptile elongation (Tan et al. 1992b). This treatment decreased the cell wall mechanical extensibility, concomitant with a substantial increase in the content of cell wall‐bound *p*‐coumaric acid in coleoptiles. These findings suggested that the accumulation of cell wall‐bound *p*‐coumaric acid modified the cell wall mechanical properties. Consequently, the accumulation of *p*‐coumaric acid in the cell wall at the interface between the epidermal and inner tissues may be attributable to the enhancement of adhesive strength between these two tissues in light‐irradiated epicotyls. Further studies employing mutant plants, inhibitors of phenylpropanoid biosynthesis, and the exogenous application of phenolic acids would help clarify this hypothesis.

Our analysis also showed that the cell walls of light‐irradiated epicotyls did not contain detectable amounts of dimeric phenolic acids (e.g., diferulic acids). This suggests that increased adhesive strength between both tissues is not attributed to the formation of cross‐linkages within cell wall architecture. On the other hand, a preliminary experiment using light‐irradiated pea epicotyls revealed the presence of some *p*‐coumaric acid in the fraction extracted from the cell wall using a hot EDTA solution. Since this fraction contains pectic polysaccharides (Sakurai et al. 1987), it is conceivable that some of the observed *p*‐coumaric acid may be associated with pectic polysaccharides. Light irradiation increased the amount of matrix polysaccharides containing pectic polysaccharides per unit length of epicotyls (Figure 5A). Masuda et al. (1981) reported an increase in the amount of pectic polysaccharides in the upper region of pea epicotyls upon light irradiation. Pectic polysaccharides are a major component of the middle lamella, a layer located within the central region of the double cell wall that is formed by two adjacent cells. Moreover, the middle lamella is involved in plant cell wall adhesion; therefore, the accumulation of *p*‐coumaric acid‐linked pectic polysaccharides may help increase the adhesive strength between plant tissues, possibly by interfering with the enzymatic degradation of polymers (Fry 1984).

The irradiation of white light resulted in a substantial increase in the content of cell wall‐bound *p*‐coumaric acid, particularly within epidermal tissue (Figure 5B). Phenolic acid monomers, *p*‐coumaric acid and ferulic acid, are synthesized via the phenylpropanoid pathway. In this pathway, phenylalanine ammonia‐lyase catalyzes the initial reaction and plays a rate‐limiting role (Deng and Lu 2017; Barros and Dixon 2020). White light irradiation enhanced the activity of phenylalanine ammonia‐lyase in maize coleoptiles, where it has been found to promote the formation of cell wall‐bound phenolic acids (Parvez et al. 1997). In addition, the transcriptional regulation of phenylalanine ammonia‐lyase genes is pivotal in modulating enzyme activity (Rohde et al. 2004; Huang et al. 2010). In a preliminary experiment, an increase in the expression level of phenylalanine ammonia‐lyase gene in pea epicotyls was observed in response to light irradiation. A comprehensive gene expression analysis in pea epidermal tissue is currently being planned. Further studies should elucidate the impact of light on the expression levels of genes encoding enzymes involved in the phenylpropanoid pathway. After formation, synthesized *p*‐coumaric acid and ferulic acid are incorporated into the cell wall. Furthermore, BAHD acyltransferases (i.e., acyl‐CoA transferases) have been found to be responsible for the incorporation of phenolic acid monomers into cell wall components in gramineous plants (Buanafina and Morris 2022; Chandrakanth et al. 2023). A recent functional analysis of BAHD acyltransferase genes in rice revealed that at least two *OsAT* genes may be responsible for the transfer of *p*‐coumaric acid residue to cell wall polysaccharides and monolignol (Bartley et al. 2013; Withers et al. 2012). Taken together, these findings suggest that the set of genes required for cell wall‐bound *p*‐coumaric acid biosynthesis may be induced by light irradiation in the epidermal tissue of pea epicotyls.

In addition to cell wall‐bound phenolic acids, the matrix polysaccharide and cellulose contents per unit epicotyl length recorded for both the epidermal and inner tissues significantly increased in response to light irradiation (Figure 5A). A previous study has shown that cell wall polysaccharide content per unit stem length represents the thickness of the cell wall (Kutschera 1990). The results obtained here suggest that there is an increase in the thickness of the cell walls of pea epicotyls under light‐grown conditions. In general, the thickening of the cell wall has been found to decrease the mechanical extensibility of the cell wall in stem organs (Kutschera 1990; Soga et al. 1999; Hattori et al. 2022; Wakabayashi et al. 2023). Consequently, thickening of the cell wall, combined with the accumulation of cell wall‐bound *p*‐coumaric acid, may cooperate to increase the adhesive strength between the epidermal and inner tissues in pea epicotyls. These findings advance our understanding of light‐regulated cell wall properties and tissue adhesion in dicotyledonous seedlings and provide a foundation for future studies on the role of wall‐bound phenolics in growth regulation and mechanical integrity.

## 5 Conclusions

In this study, we found that white light irradiation caused a substantial accumulation of cell wall‐bound *p‐*coumaric acid in epidermal tissue and in adjacent inner tissue cell layers at the second internode of pea epicotyls. This phenomenon may be responsible for increased adhesive strength between the epidermal and inner tissues. Moreover, the increased adhesive strength between both tissues may inhibit the elongation growth of pea epicotyls, possibly by limiting expansive deformation of inner tissues. Taken together, the findings presented in the study offer novel insight into the effects of light stimuli on the adhesive strength of plant tissues and on the dynamics of cell wall‐bound phenolic acids in dicotyledonous plants. Moreover, since these phenomena have not yet been reported, they may be a novel photoresponse in plants and their characterization may improve our understanding of plant photomorphogenesis. Furthermore, transgenic plants engineered to modify the levels of cell wall‐bound phenolic acids and thereby alter the adhesive strength between epidermal and inner tissues may enable precise regulation of plant growth and facilitate the modulation of stem rigidity, ultimately contributing to a broad range of industrial applications.

## Author Contributions

Y.S. and K.W. did the investigation. Y.S. did the formal analysis and the writing of the original draft. K.W. and K.M. were responsible for the supervision. K.S. did the project administration. All authors did the writing, reviewing, and editing, and approved the final manuscript.

## Funding

This work was supported by the Sasakawa Scientific Research Grant from The Japan Science Society (no. 2025‐4004) and JST SPRING (JPMJSP2139) to Y.S.

## Supporting information


**Figure S1:** ppl70755‐sup‐0001‐FigureS1.pdf.

## Data Availability

The data that support the findings of this study are available from the corresponding author upon reasonable request.

## References

[ppl70755-bib-0001] Bar, M. , and N. Ori . 2014. “Leaf Development and Morphogenesis.” Development 141, no. 22: 4219–4230. 10.1242/dev.106195. 25371359

[ppl70755-bib-0002] Barros, J. , and R. A. Dixon . 2020. “Plant Phenylalanine/Tyrosine Ammonia‐Lyases.” Trends in Plant Science 25, no. 1: 66–79. 10.1016/j.tplants.2019.09.011. 31679994

[ppl70755-bib-0003] Bartley, L. E. , M. L. Peck , S. R. Kim , et al. 2013. “Overexpression of a BAHD Acyltransferase, OsAt10, Alters Rice Cell Wall Hydroxycinnamic Acid Content and Saccharification.” Plant Physiology 161, no. 4: 1615–1633. 10.1104/pp.112.208694. 23391577 PMC3613443

[ppl70755-bib-0004] Buanafina, M. M. D. O. , and P. Morris . 2022. “The Impact of Cell Wall Feruloylation on Plant Growth, Responses to Environmental Stress, Plant Pathogens and Cell Wall Degradability.” Agronomy 12, no. 8: 1847. 10.3390/agronomy12081847.

[ppl70755-bib-0005] Chandrakanth, N. N. , C. Zhang , J. Freeman , W. R. de Souza , L. E. Bartley , and R. A. Mitchell . 2023. “Modification of Plant Cell Walls With Hydroxycinnamic Acids by BAHD Acyltransferases.” Frontiers in Plant Science 13: 1088879. 10.3389/fpls.2022.1088879. 36733587 PMC9887202

[ppl70755-bib-0006] Chen, L. , S. Kamisaka , and T. Hoson . 1999. “Breakdown of (1→3),(1→4)‐β‐d‐Glucans During Development of Rice Coleoptiles in Air and Under Water.” Journal of Plant Physiology 155, no. 2: 234–239. 10.1016/S0176-1617(99)80012-1.

[ppl70755-bib-0007] Cosgrove, D. J. 1988. “Mechanism of Rapid Suppression of Cell Expansion in Cucumber Hypocotyls After Blue‐Light Irradiation.” Planta 176, no. 1: 109–116. 10.1007/BF00392486. 24220741

[ppl70755-bib-0008] Deng, Y. , and S. Lu . 2017. “Biosynthesis and Regulation of Phenylpropanoids in Plants.” Critical Reviews in Plant Sciences 36, no. 4: 257–290. 10.1080/07352689.2017.1402852.

[ppl70755-bib-0009] DuBois, M. , K. A. Gilles , J. K. Hamilton , P. T. Rebers , and F. Smith . 1956. “Colorimetric Method for Determination of Sugars and Related Substances.” Analytical Chemistry 28, no. 3: 350–356. 10.1021/ac60111a017.

[ppl70755-bib-0010] Fry, S. C. 1984. “Incorporation of [^14^C] Cinnamate Into Hydrolase‐Resistant Components of the Primary Cell Wall of Spinach.” Phytochemistry 23, no. 1: 59–64. 10.1016/0031-9422(84)83078-2.

[ppl70755-bib-0011] Gall, H. L. , F. Philippe , J. M. Domon , F. Gillet , J. Pelloux , and C. Rayon . 2015. “Cell Wall Metabolism in Response to Abiotic Stress.” Plants 4, no. 1: 112–166. 10.3390/plants4010112. 27135320 PMC4844334

[ppl70755-bib-0012] Harris, P. J. , and R. D. Hartley . 1976. “Detection of Bound Ferulic Acid in Cell Walls of the Gramineae by Ultraviolet Fluorescence Microscopy.” Nature 259, no. 5543: 508–510. 10.1038/259508a0.

[ppl70755-bib-0013] Harris, P. J. , and R. D. Hartley . 1980. “Phenolic Constituents of the Cell Walls of Monocotyledons.” Biochemical Systematics and Ecology 8, no. 2: 153–160. 10.1016/0305-1978(80)90008-3.

[ppl70755-bib-0014] Hartley, R. D. , and P. J. Harris . 1981. “Phenolic Constituents of the Cell Walls of Dicotyledons.” Biochemical Systematics and Ecology 9, no. 2–3: 189–203. 10.1016/0305-1978(81)90040-5.

[ppl70755-bib-0015] Hattori, T. , K. Soga , K. Wakabayashi , and T. Hoson . 2022. “An Arabidopsis *PTH2* Gene Is Responsible for Gravity Resistance Supporting Plant Growth Under Different Gravity Conditions.” Life 12, no. 10: 1603. 10.3390/life12101603. 36295039 PMC9605376

[ppl70755-bib-0016] Huang, J. , M. Gu , Z. Lai , et al. 2010. “Functional Analysis of the Arabidopsis *PAL* Gene Family in Plant Growth, Development, and Response to Environmental Stress.” Plant Physiology 153, no. 4: 1526–1538. 10.1104/pp.110.157370. 20566705 PMC2923909

[ppl70755-bib-0017] Karimali, D. , I. Kosma , and A. Badeka . 2020. “Varietal Classification of Red Wine Samples From Four Native Greek Grape Varieties Based on Volatile Compound Analysis, Color Parameters and Phenolic Composition.” European Food Research and Technology 246, no. 1: 41–53. 10.1007/s00217-019-03398-7.

[ppl70755-bib-0018] Kelly‐Bellow, R. , K. Lee , R. Kennaway , et al. 2023. “Brassinosteroid Coordinates Cell Layer Interactions in Plants via Cell Wall and Tissue Mechanics.” Science 380, no. 6651: 1275–1281. 10.1126/science.adf0752. 37347863

[ppl70755-bib-0019] Kigel, J. , and D. J. Cosgrove . 1991. “Photoinhibition of Stem Elongation by Blue and Red Light: Effects on Hydraulic and Cell Wall Properties.” Plant Physiology 95, no. 4: 1049–1056. 10.1104/pp.95.4.1049. 11537486 PMC1077650

[ppl70755-bib-0020] Kutschera, U. 1990. “Cell‐Wall Synthesis and Elongation Growth in Hypocotyls of *Helianthus annuus* L.” Planta 181, no. 3: 316–323. 10.1007/bf00195882. 24196808

[ppl70755-bib-0021] Kutschera, U. , R. Bergfeld , and P. Schopfer . 1987. “Cooperation of Epidermis and Inner Tissues in Auxin‐Mediated Growth of Maize Coleoptiles.” Planta 170, no. 2: 168–180. 10.1007/bf00397885. 24232875

[ppl70755-bib-0022] Kutschera, U. , and K. J. Niklas . 2007. “The Epidermal‐Growth‐Control Theory of Stem Elongation: An Old and a New Perspective.” Journal of Plant Physiology 164, no. 11: 1395–1409. 10.1016/j.jplph.2007.08.002. 17905474

[ppl70755-bib-0023] Masuda, Y. , S. Kamisaka , H. Yanagisawa , and Y. Suzuki . 1981. “Effect of Light on Growth and Metabolic Activities in Pea Seedlings I. Changes in Cell Wall Polysaccharides During Growth in the Dark and in the Light.” Biochemie und Physiologie der Pflanzen 176, no. 1: 23–34. 10.1016/S0015-3796(81)80005-4.

[ppl70755-bib-0024] Masuda, Y. , and R. Yamamoto . 1972. “Control of Auxin‐Induced Stem Elongation by the Epidermis.” Physiologia Plantarum 27, no. 2: 109–115. 10.1111/j.1399-3054.1972.tb03585.x.

[ppl70755-bib-0025] Miyamoto, K. , Y. Mitani , K. Soga , et al. 1997. “Modification of Chemical Properties of Cell Wall Polysaccharides in the Inner Tissues by White Light in Relation to the Decrease in Tissue Tension in *Pisum sativum* Epicotyls.” Physiologia Plantarum 101, no. 1: 38–44. 10.1111/j.1399-3054.1997.tb01817.x.

[ppl70755-bib-0026] Miyamoto, K. , J. Ueda , T. Hoson , S. Kamisaka , and Y. Masuda . 1992. “Inhibition of *Pisum sativum* Epicotyl Elongation by White Light – Different Effects of Light on the Mechanical Properties of Cell Walls in the Epidermal and Inner Tissues.” Physiologia Plantarum 84, no. 3: 380–385. 10.1111/j.1399-3054.1992.tb04679.x.

[ppl70755-bib-0027] Moura, J. C. M. S. , C. A. V. Bonine , J. de Oliveira Fernans Viana , M. C. Dornelas , and P. Mazzafera . 2010. “Abiotic and Biotic Stresses and Changes in the Lignin Content and Composition in Plants.” Journal of Integrative Plant Biology 52, no. 4: 360–376. 10.1111/j.1744-7909.2010.00892.x. 20377698

[ppl70755-bib-0028] Parvez, M. M. , K. Wakabayashi , T. Hoson , and S. Kamisaka . 1996. “Changes in Cellular Osmotic Potential and Mechanical Properties of Cell Walls During Light‐Induced Inhibition of Cell Elongation in Maize Coleoptiles.” Physiologia Plantarum 96, no. 2: 179–185. 10.1111/j.1399-3054.1996.tb00199.x.

[ppl70755-bib-0029] Parvez, M. M. , K. Wakabayashi , T. Hoson , and S. Kamisaka . 1997. “White Light Promotes the Formation of Diferulic Acid in Maize Coleoptile Cell Walls by Enhancing PAL Activity.” Physiologia Plantarum 99, no. 1: 39–48. 10.1111/j.1399-3054.1997.tb03428.x.

[ppl70755-bib-0030] Richtzenhain, H. 1949. “Oestrogen stilben‐ und diphenyläthan‐derivate, II. Mitteilung.” Chemische Berichte 82, no. 4–5: 405–407. 10.1002/cber.19490820416.

[ppl70755-bib-0031] Rohde, A. , K. Morreel , J. Ralph , et al. 2004. “Molecular Phenotyping of the *pal1* and *pal2* Mutants of *Arabidopsis thaliana* Reveals Far‐Reaching Consequences on Phenylpropanoid, Amino Acid, and Carbohydrate Metabolism.” Plant Cell 16, no. 10: 2749–2771. 10.1105/tpc.104.023705. 15377757 PMC520969

[ppl70755-bib-0032] Sakurai, N. , S. Tanaka , and S. Kuraishi . 1987. “Changes in Wall Polysaccharides of Squash (*Cucurbita maxima* Duch.) Hypocotyls Under Water Stress Condition: I. Wall Sugar Composition and Growth as Affected by Water Stress.” Plant and Cell Physiology 28, no. 6: 1051–1058. 10.1093/oxfordjournals.pcp.a077385.

[ppl70755-bib-0033] Shimizu, Y. , K. Wakabayashi , K. Miyamoto , and K. Soga . 2025. “Measurement of Adhesive Strength Between the Epidermal and Inner Tissues of Plant Stems Using a Tensile Tester.” Biorheology 60, no. 1–2: 23–30. 10.1177/0006355X241296333. 40392015

[ppl70755-bib-0034] Soga, K. , K. Wakabayashi , T. Hoson , and S. Kamisaka . 1999. “Hypergravity Increases the Molecular Mass of Xyloglucans by Decreasing Xyloglucan‐Degrading Activity in Azuki Bean Epicotyls.” Plant and Cell Physiology 40, no. 6: 581–585. 10.1093/oxfordjournals.pcp.a029580. 10483122

[ppl70755-bib-0035] Symons, G. M. , and J. B. Reid . 2003. “Interactions Between Light and Plant Hormones During de‐Etiolation.” Journal of Plant Growth Regulation 22, no. 1: 3–14. 10.1007/s00344-003-0017-8.

[ppl70755-bib-0036] Tan, K. S. , T. Hoson , Y. Masuda , and S. Kamisaka . 1992a. “Involvement of Cell Wall‐Bound Diferulic Acid in Light‐Induced Decrease in Growth Rate and Cell Wall Extensibility of *Oryza* Coleoptiles.” Plant and Cell Physiology 33, no. 2: 103–108. 10.1093/oxfordjournals.pcp.a078227.

[ppl70755-bib-0037] Tan, K. S. , T. Hoson , Y. Masuda , and S. Kamisaka . 1992b. “Effect of Ferulic and *p‐*Coumaric Acids on *Oryza* Coleoptile Growth and the Mechanical Properties of Cell Walls.” Journal of Plant Physiology 140, no. 4: 460–465. 10.1016/S0176-1617(11)80825-4.

[ppl70755-bib-0038] Waadt, R. , C. A. Seller , P. K. Hsu , Y. Takahashi , S. Munemasa , and J. I. Schroeder . 2022. “Plant Hormone Regulation of Abiotic Stress Responses.” Nature Reviews Molecular Cell Biology 23, no. 10: 680–694. 10.1038/s41580-022-00479-6. 35513717 PMC9592120

[ppl70755-bib-0039] Wakabayashi, K. , T. Hoson , and N. Sakurai . 1999. “Auxin Stimulates the Synthesis but Not the Loosening of Cell Walls in Isolated Outer Tissue of Dark‐Grown Squash (*Cucurbita maxima* Duch.) Hypocotyls.” Journal of Plant Physiology 154, no. 2: 197–202. 10.1016/S0176-1617(99)80210-7.

[ppl70755-bib-0040] Wakabayashi, K. , S. Nakano , K. Soga , and T. Hoson . 2009. “Cell Wall‐Bound Peroxidase Activity and Lignin Formation in Azuki Bean Epicotyls Grown Under Hypergravity Conditions.” Journal of Plant Physiology 166, no. 9: 947–954. 10.1016/j.jplph.2008.12.006. 19195738

[ppl70755-bib-0041] Wakabayashi, K. , N. Sakurai , and S. Kuraishi . 1991. “Differential Effect of Auxin on Molecular Weight Distributions of Xyloglucans in Cell Walls of Outer and Inner Tissues From Segments of Dark‐Grown Squash (*Cucurbita maxima* Duch.) Hypocotyls.” Plant Physiology 95, no. 4: 1070–1076. 10.1104/pp.95.4.1070. 16668092 PMC1077653

[ppl70755-bib-0042] Wakabayashi, K. , K. Soga , T. Hoson , et al. 2015. “Suppression of Hydroxycinnamate Network Formation in Cell Walls of Rice Shoots Grown Under Microgravity Conditions in Space.” PLoS One 10, no. 9: e0137992. 10.1371/journal.pone.0137992. 26378793 PMC4574559

[ppl70755-bib-0043] Wakabayashi, K. , K. Soga , T. Hoson , and H. Masuda . 2023. “The Modification of Cell Wall Properties Is Involved in the Growth Inhibition of Rice Coleoptiles Induced by Lead Stress.” Life 13, no. 2: 471. 10.3390/life13020471. 36836828 PMC9967465

[ppl70755-bib-0044] Waldron, K. W. , A. J. Parr , A. Ng , and J. Ralph . 1996. “Cell Wall Esterified Phenolic Dimers: Identification and Quantification by Reverse Phase High Performance Liquid Chromatography and Diode Array Detection.” Phytochemical Analysis 7, no. 6: 305–312. 10.1002/(SICI)1099-1565(199611)7:6<305::AID-PCA320>3.0.CO;2-A.

[ppl70755-bib-0045] Withers, S. , F. Lu , H. Kim , Y. Zhu , J. Ralph , and C. G. Wilkerson . 2012. “Identification of Grass‐Specific Enzyme That Acylates Monolignols With *p‐*Coumarate.” Journal of Biological Chemistry 287, no. 11: 8347–8355. 10.1074/jbc.m111.284497. 22267741 PMC3318722

[ppl70755-bib-0046] Zhou, R. , G. Liu , D. Li , et al. 2023. “An Advanced Organic Molecular Probe for Multimodal Fluorescence Imaging of Cellular Lipid Droplets.” Sensors and Actuators B: Chemical 387: 133772. 10.1016/j.snb.2023.133772.

