# Supplementary material for: White Light Enhances Adhesive Strength Between Epidermal and Inner Tissues of Pea Epicotyls via Accumulation of Cell Wall‐Bound p‐Coumaric Acid
Source: Physiol Plant. 2026 Jan 25;178(1):e70755. doi: 10.1111/ppl.70755 (PMC12832603; doi:10.1111/ppl.70755)
Supplement: Supplementary file 1 — Figure S1: ppl70755‐sup‐0001‐FigureS1.pdf. [file PPL-178-e70755-s001.pdf]

White light enhances adhesive strength between epidermal and inner tissues of pea epicotyls via accumulation of cell wall-bound *p*-coumaric acid

Yuma Shimizu, Kazuyuki Wakabayashi, Kensuke Miyamoto, Kouichi Soga

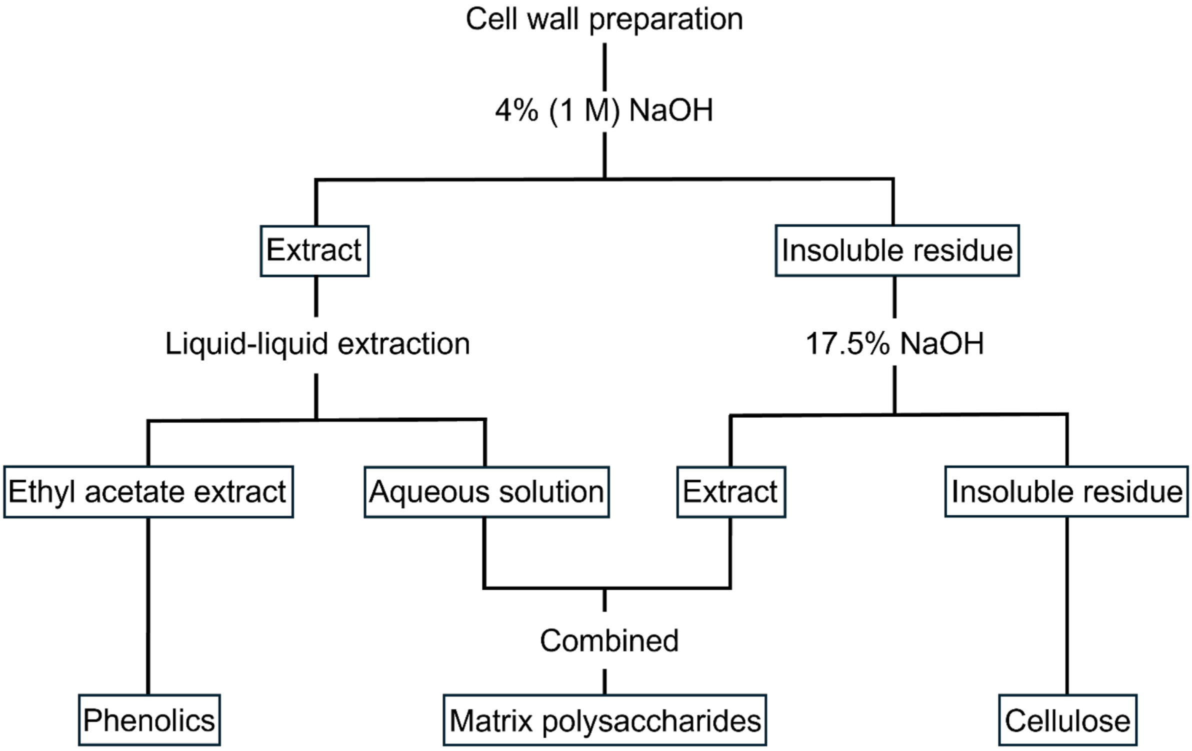

Figure S1: Sequential extraction of cell wall components from cell wall preparation. Ester-linked phenolic acids (phenolics) were extracted with NaOH (4% w/v, 1 M), and matrix polysaccharides were obtained by combining extracts from 4% and 17.5% (w/v) NaOH. The remaining alkali-insoluble residue was designated as the cellulose fraction. The liberated phenolic acids were analyzed using an HPLC equipped with a reversed-phase column.
